# Determining Whether Tai Chi Chuan Is Related to the Updating Function in Older Adults: Differences Between Practitioners and Controls

**DOI:** 10.3389/fpubh.2022.797351

**Published:** 2022-05-03

**Authors:** Yuan Yang, Tingting Chen, Chen Wang, Ji Zhang, Xiaoxia Yuan, Xiaoke Zhong, Shoufu Yan, Changhao Jiang

**Affiliations:** ^1^College of Physical Education and Sports, Beijing Normal University, Beijing, China; ^2^School of Education, Beijing Dance Academy, Beijing, China; ^3^The Center of Neuroscience and Sports, Capital University of Physical Education and Sports, Beijing, China; ^4^Beijing Key Laboratory of Physical Fitness Evaluation and Technical Analysis, Capital University of Physical Education and Sports, Beijing, China; ^5^School of Kinesiology and Health, Capital University of Physical Education and Sports, Beijing, China

**Keywords:** updating function, Tai Chi Chuan, older adults, Reaction Times (RTs), Accuracy Rates (ARs)

## Abstract

**Background:**

Tai Chi Chuan (TCC) is an effective method for delaying cognitive decline in older adults. However, in older adults, the association between long-term TCC practice and working memory updating has not been extensively studied.

**Objective:**

This cross-sectional study investigated how updating function operationalized *via* Reaction Times (RTs) and Accuracy Rates (ARs) of N-Back tasks being measured in a laboratory setting is related to long term practice of TCC.

**Methods:**

Twenty-six healthy elderly people participated in this experiment. According to the duration of time TCC was practiced, 13 subjects in the TCC group had more than 5 years of experience with TCC exercise, and 13 elderly subjects who had not been systematically exposed to mind-body exercise were assigned to the control group. The N-back task was administered to every participant to evaluate the updating function.

**Results:**

The TCC group had faster RTs than the control group (*p* < 0.05). For the 1-back task, the TCC group showed faster RTs than the control group; for the 2-back task, the TCC group exhibited faster RTs than the control group. The TCC group had higher ARs than the control group (*p* < 0.05). For the 1-back task, the TCC group showed higher ARs than the control group; for the 2-back task, the TCC group exhibited higher ARs than the control group.

**Conclusions:**

Long-term TCC practitioners exhibit a better updating function as compared to controls who did not practice TCC. Thus, our findings suggest that long-term TCC positively influences the updating function of older adults, making it, in turn, an effective mind-body exercise to maintain specific aspects of cognitive functioning.

## Introduction

The increase in the aging population and the subsequent rise in age-related diseases is a major challenge for the resources of health care systems. Elderly persons are vulnerable to a decline in physical function and a high incidence of disease (1). In addition to the decline in physical ability, the decline in cognitive functioning seriously impairs the quality of life in older adults (2). Previous studies have shown that cognitive decline associated with age-induced neurodegeneration and altered brain activation patterns compared to younger adults (3–6).

Executive function decline is one of the manifestations of cognitive decline. Executive function refers to the coordination of various resources and the processing required by the control system to accomplish specific goals when the brain completes complex cognitive tasks (7). Executive function includes working memory (WM), inhibitory control (IC) and cognitive flexibility (CF) (8). Updating is part of WM, and plays an important role in the whole cognition process (9). With age, the updating function of the elderly gradually declines. N-back is a behavioral experimental paradigm for detecting updating function, and is widely used in neurocognitive research (10). There is evidence in the literature that working memory performance (assessed via the n-back task) is sensitive to age-related changes (11).

Several studies have demonstrated that a serious decline in WM occurs in neurological diseases such as Alzheimer's disease (12–14). It is expected that there will be 75 million elderly people suffering from dementia globally by 2,030 (15). Therefore, the discovery of effective measures to intervene in aging and delay WM decline has become the focus of current research in the cognitive neuroscience field. Both physiological impact and psychological benefits should be considered when choosing maintenance strategies (16).

Mindfulness has a positive effect on the executive function of elderly individuals and may be beneficial for the WM performance in older people (17). Tai Chi Chuan (TCC) is accompanied by concentration and breathing control to improve physical strength, balance, flexibility and promote physical health (18–20). TCC is gentle, slow, coherent, relaxed and involves stretching. Compared with other training methods that emphasize endurance and strength, TCC is more suitable for the elderly to participate (21).

There is evidence in the literature that TCC practice can improve cognitive performance in older adults with and without cognitive impairments (22, 23). Studies by Mortimer et al. (24), showed that healthy elderly people who completed 40 weeks of TCC practice performed better in working memory tasks and demonstrated higher activation levels of brain areas related to cognitive function. Lam et al. (25), randomly allocated elderly subjects to experimental and control groups in which the experimental group was required to complete TCC exercise three times a week, for 30 min each time, for 1 year, while the control group only performed stretching exercises. After 5 months of exercise, the test scores of the experimental group's working memory task improved, and continued to improve, after 1 year.

Although many studies have verified the effect of TCC on improving WM, the results have been inconsistent. For example, a 20 weeks TCC intervention study conducted by Hall et al. (26), showed that there was no significant change in the behavioral performance of elderly individuals in the cognitive task after TCC intervention. However, studies have found that mindfulness meditation exercise has a specific effect on executive function, the most specific inhibitory component, and the findings concerning updating and shifting domains are heterogeneous (27, 28). It is unclear whether the benefit of mindfulness in improving executive function is a selective improvement of one subability or an improvement of the whole. At present, the effect of mindfulness on the results is inconsistent, and needs to be further verified. Zeidan et al. (29) indicated that brief mindfulness training improves N-back task performance and has favorable effects on the number of correct responses over time compared to the control group. Thus, based on the specific effects of brief mindfulness, this type of intervention could improve updating function, and elevate sustained accuracy over time. Based on this result, it is necessary to investigate the relationship between long-term mindfulness training and executive function specific subability.

To address these knowledge gaps, we conducted this study to explore the possible characteristics of TCC, an oriental mindfulness exercise, in improving the updating function of aging elderly people. A cross-sectional study design and a behavioral experiment (N-back) were used to examine the relationship between TCC and the updating function of elderly individuals.

Collectively, there is evidence that TCC can improve working memory. Furthermore, it has also been observed that the practice of mindfulness exercises that are an integral part of TCC is beneficial for executive function. Based on this evidence, the two following hypotheses are proposed:

### Hypothesis 1 (H1)

TCC practitioners exhibit greater updating function than age-matched and gender-matched, non-sedentary, TCC controls.

### Hypothesis 2 (H2)

There is a positive correlation between long-term TCC and the updating function. With the increasing in the TCC training period, the working memory updating function is also enhanced.

## Materials and Methods

### Participants

The sample size was estimated based on a similar experimental design (30). G^*^Power 3.1 software (G^*^Power Software Inc., Kiel, Germany) was used to calculate the required sample size. We set the effect of *f*^2^ = 0.30, according to the 2 × 2 experimental design, which is a medium sized effect t (31, 32), adopting an alpha of 0.05 and power of 0.80, and we obtained the estimated total sample size to be 24. We recruited 26 older females from the local community from China. The screening conditions were as follows: (1) no brain trauma, psychiatric history, or chronic diseases; (2) educational background of high school and above; (3) right-handed; (4) normal vision or corrected vision; (5) a Minimum Mental State Examination (MMSE) score ≥24; (6) exercised 1 h a day. All the subjects were required to abstain from alcohol, staying up late or taking part in heavy exercise 1 day prior to the experiment, to ensure adequate sleep.

Two groups of subjects were selected according to TCC experience. Thirteen subjects in the TCC group had more than 5 years of TCC exercise experience (60 min per session for 3 days per week; average age of 65.23 ± 1.48 years; TCC for 5.77 ± 0.73 years); Thirteen elderly subjects who had not been systematically exposed to mind-body exercise were assigned to the control group (average age of 65.15 ± 3.69).

**Figure 1:**
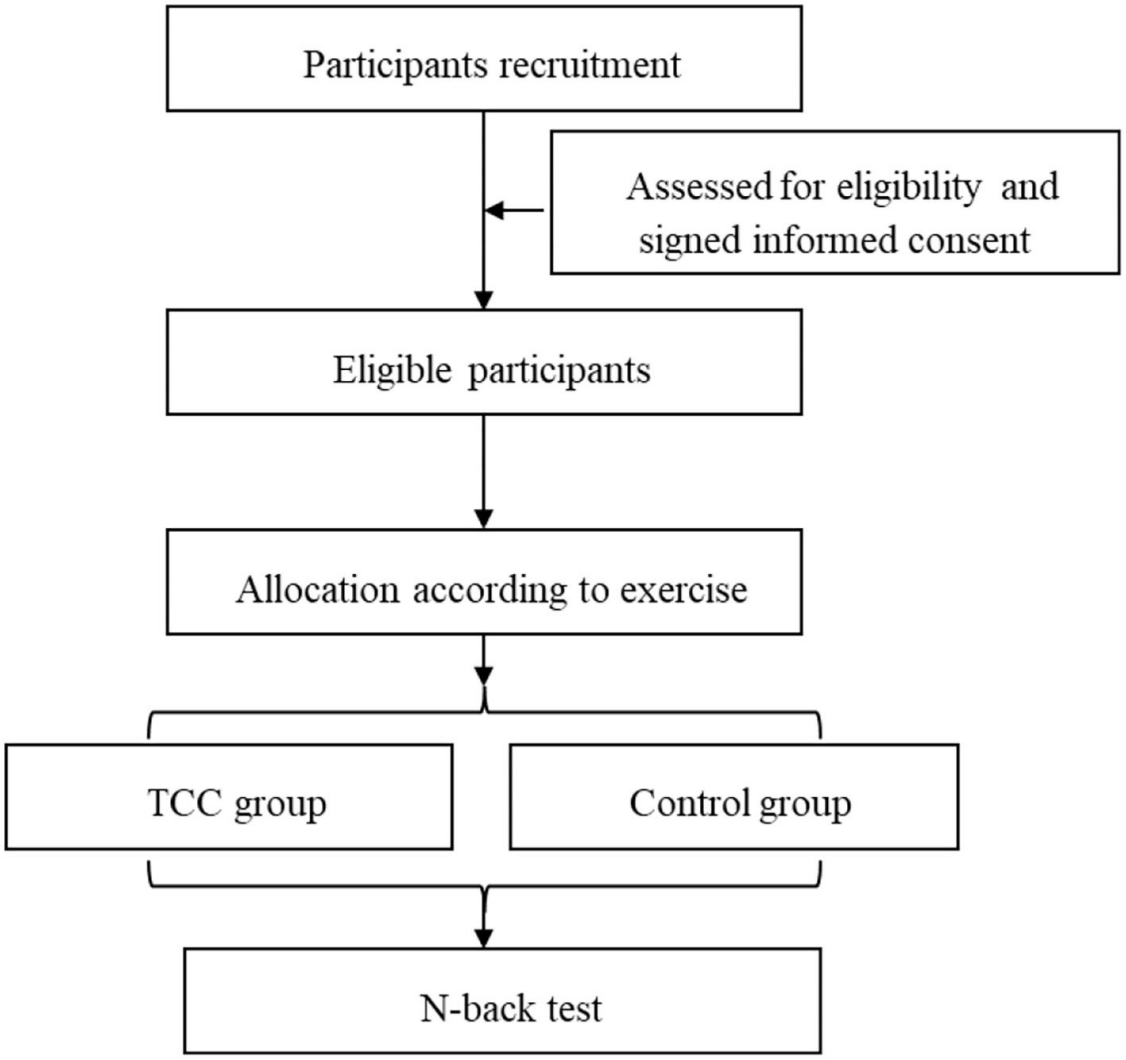
Flow diagram of the study design.

The Capital University of Physical Education and Sports (CUPES) Ethics Committee (approval No. CUPES-2018-06-15-01) approved the study, and all participants signed an informed consent form before participating in the experiment. The experimental process is shown in Figure 1.

### Procedures

**Figure 2:**
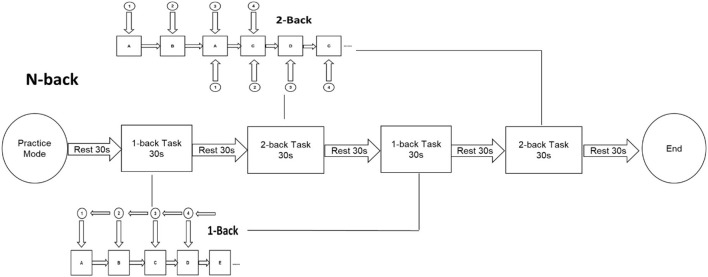
N-back task flow chart.

**Figure 3:**
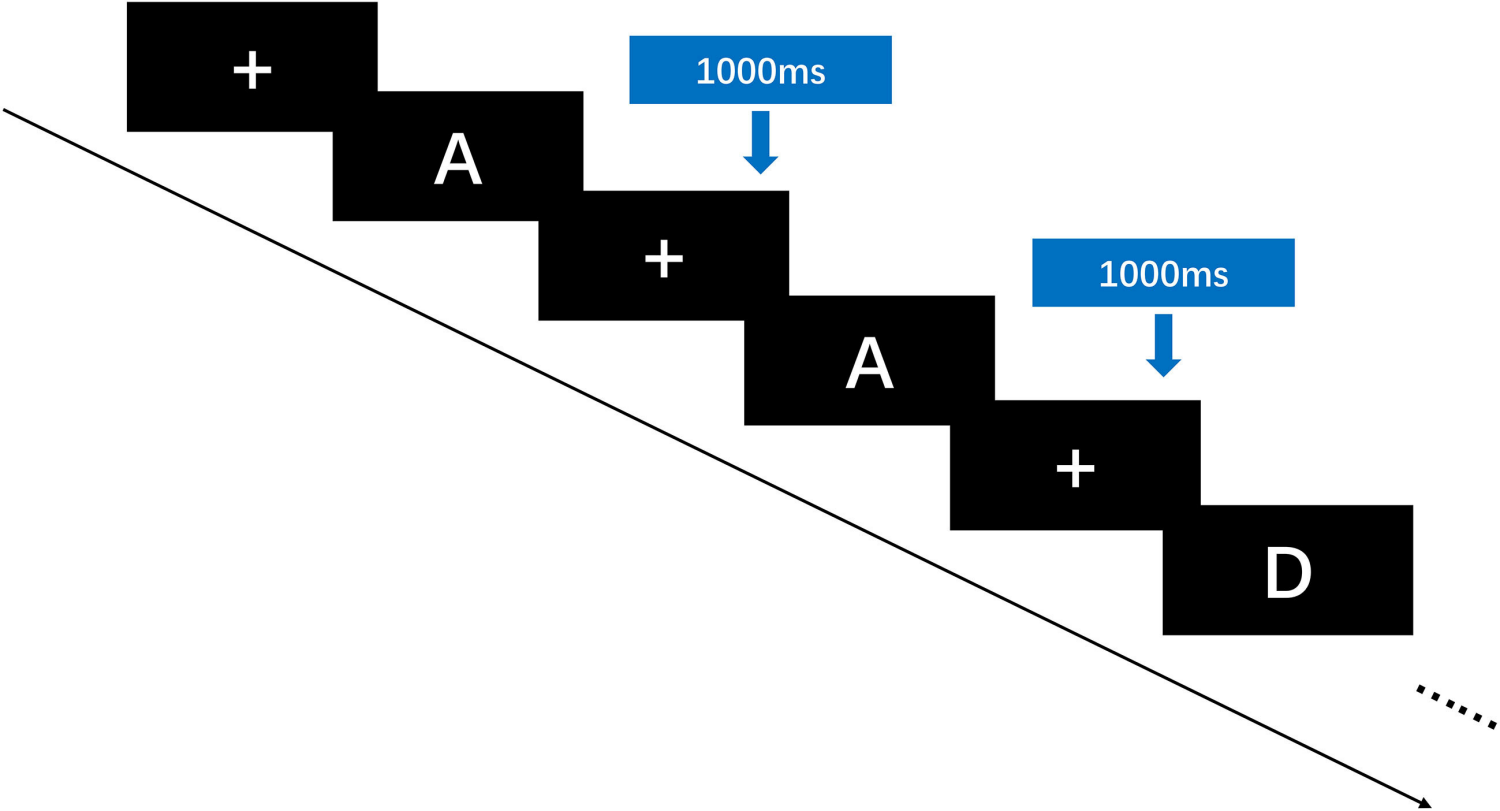
In the 1-back task, participants need to determine whether a letter appears the same as the one before it. In the 2-back task, participants need to determine whether a letter appears the same as the two before it. If the same, press “F”; if different, press “J”. The maximum reaction time is 1000 ms.

This experiment ran the N-back task paradigm using E-prime 2.0 on a 14-inch notebook computer (Surface Laptop, Microsoft) with 1024 × 768 resolution, and compiled the N-back experiment task performance with E-prime software. An N-back task paradigm was used to trial the updating function. Before the formal experiment began, the subjects were instructed to operate the practice mode according to the required proficiency task test procedures. When the accuracy rate reaches more than 85%, the formal test was allowed to start. The formal test begins at the end of the exercise mode. The N-back formal test consisted of four blocks, including two 1-back tasks and two 2-back tasks. Each block consisted of a total of 30 tests, and stimulus materials were randomly presented in the center of the computer screen. The stimulus materials for this experiment were English letters (including upper and lower cases). In each experiment, a black screen was used for key feedback. The maximum reaction time was 1,000 ms before the next stimulus cycle began. In the 1-back task, participants were required to compare the last letter with the previous one in succession. If the latter letter was the same as the previous letter, they were instructed to press the “F” key, and if different, they were instructed to press the “J” key. In the 2-back task, participants were required to compare the second letter in succession with the second letter in front. If these letters were the same, they were instructed to press the “F” key, and if different, they were instructed to press the “J” key. The entire formal test time was approximately 5 min, and subjects were given 30 s of rest time between each block; 150 s of resting time was granted overall. The entire test was carried out in a laboratory environment with quiet surroundings, and the distance to the stimulus computer was standardized. The main outcome was the reaction times (RTs) and Accuracy Rates (ARs) in performing N-back tasks. Figures 2, 3 illustrate the testing process of the N-back task.

### Statistical Analysis

The experiment was designed as a mixed-model experiment of 2 group types (TCC group, control group) × 2 task types (1-back, 2-back), in which group type was the intergroup variable, task type was the intragroup variable, and the RTs and ARs were dependent variables. IBM SPSS (Version 23, Chicago, Illinois, USA) was used for statistical analysis. Based on the presence of a normal distribution, which was verified by the Shapiro–Wilk test, we analyzed the RTs and ARs data using ANOVA. The RTs and ARs of the behavioral data were analyzed by two-way mixed ANOVA of 2 group types (TCC group, control group) × 2 task types (1-back, 2-back). If there was a significant interaction, simple effect analysis was used for further statistical analysis, and a Bonferroni correction for multiple comparisons was applied. Partial eta-squared (η2) was reported, being classified as: “small” from 0.01 to 0.06, “medium” from 0.06 to 0.14, and “large” higher than 0.14. The *p*-value was corrected by the Greenhouse-Geisser method, and the significance level α was set at a value of 0.05.

## Results

### Demographic Data Results

Before the experiment, the demographic variables of the two groups were analyzed (Table 1). They showed no significant differences in age, years of education, MMSE scores, or body mass index (BMI) among the three groups (*p* > 0.05), indicating homogeneous demographic characteristics between the groups.

**Table 1:** Demographic data of the participants (M ± SD).

**Factor**	**TCC group**	**Control group**	***p***
Age (years)	65.23 ± 1.48	65.15 ± 3.69	0.95
BMI	24.45 ± 1.34	24.16 ± 1.26	0.58
Education (years)	12.92 ± 1.76	13.54 ± 2.03	0.42
MMSE score	27.08 ± 0.86	26.77 ± 1.67	0.45

*TCC, Tai Chi Chuan group*.

### RTs Analysis of the N-Back Task

The RTs of the N-back task are shown in Table 2. When analyzing whether TCC exercise affects the updating function of individuals, the RTs of the N-back task were tested as a dependent variable. The results showed that the main effect of task type (1-back and 2-back task) was significant [F_(1, 24)_ = 27.320, *p* = 0.000, η^2^= 0.532], and the main effect of the groups (TCC group, Control group) was significant, indicating that there were differences in RTs between the two groups [F_(1, 24)_ = 16.542, *p* = 0.000,η^2^= 0.408], and the interaction between group and task type was significant [F_(1, 24)_ = 7.359, *p* = 0.012, η^2^= 0.235].

**Table 2:** N-back task RTs of participants (M ± SD).

**Task**	**TCC**	**Control**	***n***
1-back	396.06 ± 49.22^*^	429.18 ± 27.92	13
2-back	418.91 ± 44.60^*^	501.36 ± 47.00	13

*TCC, Tai Chi Chuan; RTs, Reaction times*;

* *p < 0.05*.

**Figure 4:**
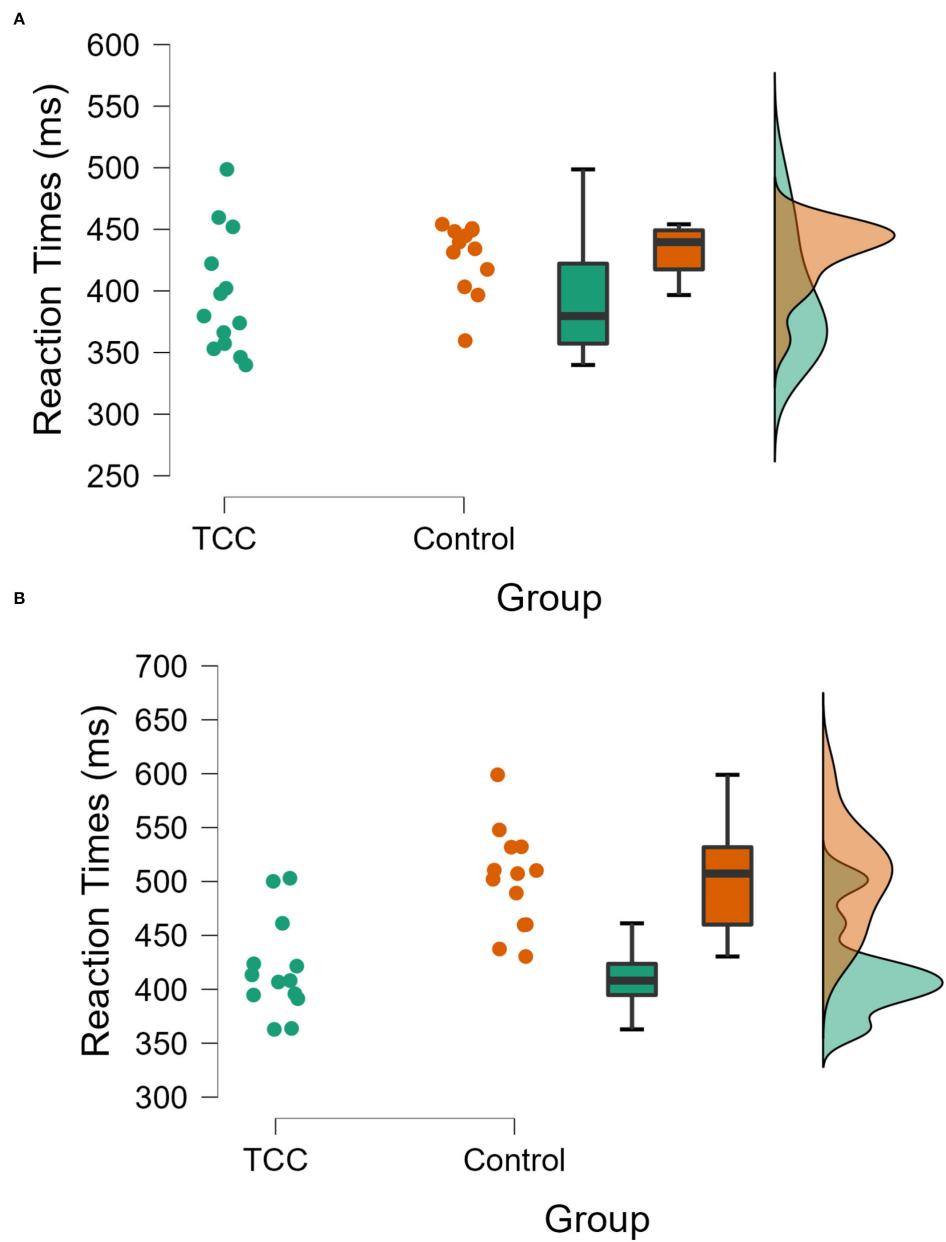
Comparison of RTs between the two groups (TCC vs. controls) during the N-back task (**A**: 1-back; **B**: 2-back).

Furthermore, the simple-effects analysis showed that under the 1-back task conditions, the RTs in the TCC group were faster than those in the control group, and the difference was significant (*p* < 0.05); the 2-back task RTs in the TCC group were faster than those in the control group, and the difference was significant (*p* < 0.05) (Figure 4). In summary, long-term TCC practitioners showed faster RTs in the 1-back and 2-back conditions.

### ARs Analysis of N-Back Task

The ARs of the N-back task are shown in Table 3. When analyzing whether TCC exercise affects the updating function of individuals, the ARs of the N-back task were tested as a dependent variable. The results showed that the main effect of task type (1-back and 2-back) was significant [F_(1, 24)_ = 10.381, *p* = 0.004, η^2^= 0.302], and the main effect of group (TCC group, control group) was significant [F_(1, 24)_ = 26.868, *p* = 0.000, η^2^= 0.528]. However, the interaction between groups and task type was not significant [F_(1, 24)_ = 0.106, *p* = 0.747, η^2^= 0.004].

**Table 3:** N-back task ARs of participants (M ± SD).

**Task**	**TCC**	**Control**	***n***
1-back	0.69 ± 0.06^*^	0.56 ± 0.04	13
2-back	0.64 ± 0.11^*^	0.52 ± 0.06	13

*TCC, Tai Chi Chuan; ARs, Accuracy Rates*;

* *p < 0.05*.

**Figure 5:**
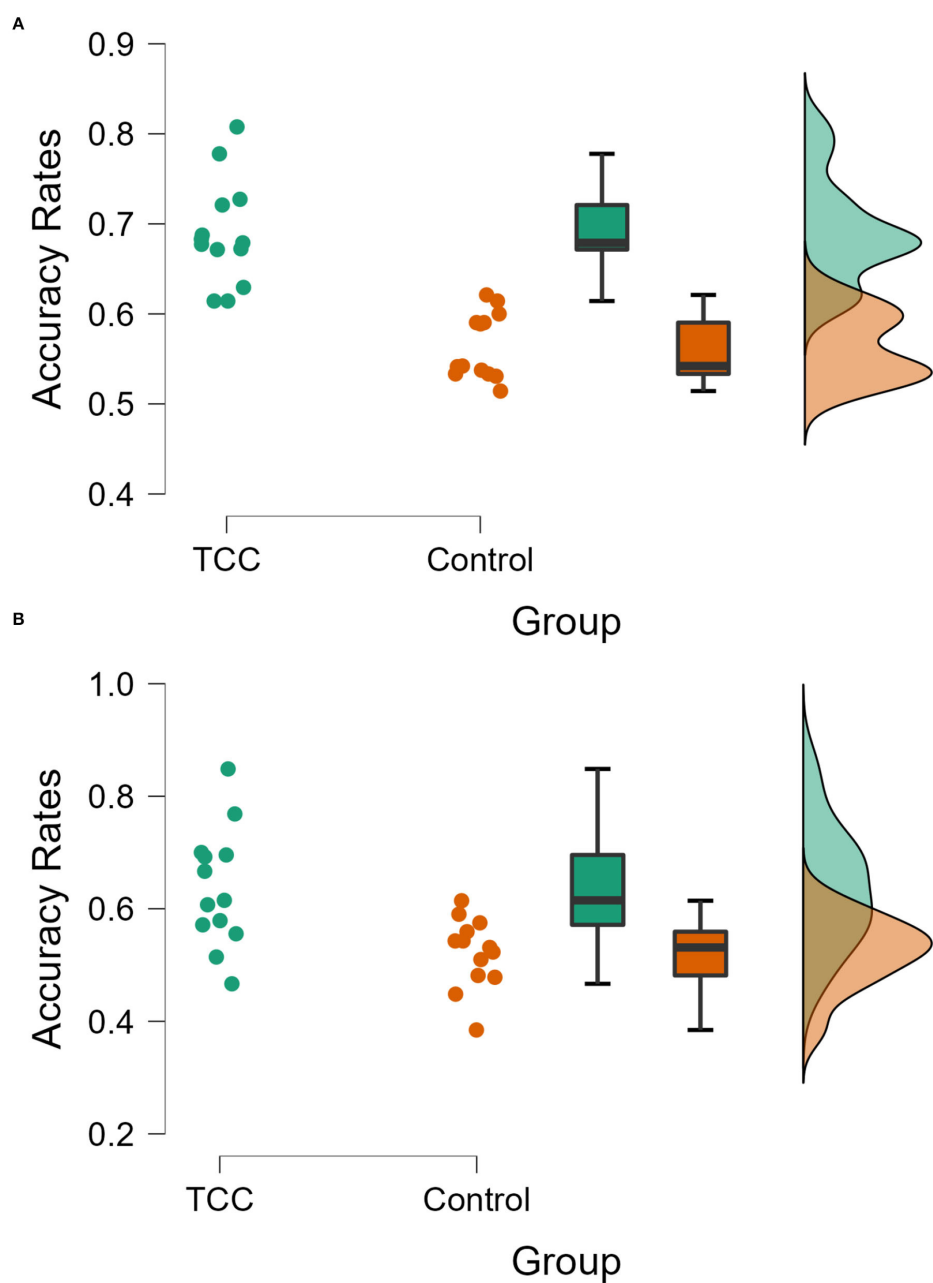
Comparison of ARs between the two groups (TCC vs. controls) during the N-back task (**A**: 1-back; **B**: 2-back).

The *post hoc* comparison showed that in the 1-back task, the ARs of the TCC group were significantly higher than those of the control group (0.69 vs. 0.56, *p* < 0.001). In the 2-back task, the ARs of the TCC group were significantly higher than those of the control group (0.64 vs. 0.52, *p* = 0.001) (Figure 5). In summary, long-term TCC practitioners showed higher ARs in the 1-back and 2-back conditions.

## Discussion

This study aimed to examine the long-term effects of TCC exercise on the updating aspect of WM performance in older adults in behavioral domains between practitioners and controls. The results showed that compared with the control group, the TCC group had faster RTs in completing the N-back task, suggesting that TCC could promote updating function in elderly individuals. This result is consistent with the existing literature. In the study of Man and colleagues (33), 135 elderly subjects were assigned to the Tai Chi group, the general exercise group or the control group, and the WM of the subjects was examined by relevant behavioral tasks. The results showed that the subjects who habitually performed Tai Chi exercise had better WM.

The present study shows that the long-term practice of TCC can improve the working memory updating function of elderly individuals. We also considered the psychological and neural mechanisms of the cognitive benefits of this exercise, taking into account the characteristics of the N-back task and TCC. Combined with the behavioral data in this study, it was found that compared with the 2-back task, the RTs of the subjects in the 1-back task were significantly lower, which indicated that subjects needed to mobilize more cognitive resources to process more complex information. These cognitive performances can be improved through exercise and cognitive training. Therefore, a long-term TCC may improve the updating function (16).

Previous neuroimaging studies showed that TCC interventions can reshape the human brain structure and can lead to alterations of functional brain patterns, which, in turn, might foster the improvement of cognitive performance (34). For instance, in the study of Yue and colleagues (35), 42 healthy elderly women were assigned to two groups (TCC group and walking group) and underwent a cognitive assessment, as well as a functional magnetic resonance imaging to quantify between-group differences in brain structure and resting-state functional connectivity. In the above-mentioned study, it was noticed (i) that episodic memory in the TCC group was superior to that of the walking group, (ii) that the TCC group exhibited a higher gray matter density in the inferior and medial temporal regions and higher values of regional homogeneity in temporal regions as compared to the walking groups, and (iii) that neurobehavioural relationships exist (i.e., the correlation between gray matter density of the left hippocampus and episodic memory performance). Another magnetic resonance imaging study by Yue et al. (36) revealed that regular TCC practice can improve measures of brain functioning and networking of elderly individuals (i.e., operationalized by graph-based metrics). Yang and colleagues (16) recruited 26 older adults to participate in a clinical trial and randomly allocated them either to the TCC group or the control group. The older adults in the TCC group trained for 8 weeks, whereas the control group was advised to maintain their general level of regular physical activity, Yang and colleagues observed that TCC intervention improved the performance of inhibitory control (i.e., operationalized by reaction times in Flanker task) that was accompanied by increased activation of the left frontal lobe (i.e., operationalized *via* functional near-infrared spectroscopy). Taken together these findings suggest that TCC interventions can positively influence brain structure, brain function, and cognitive performance in older adults.

TCC is an oriental mindfulness exercise that integrates flexibility and coordination, and its exercise mechanisms are closely related to the WM of elderly individuals (37). It requires the participant to complete cognitive activities, such as visual space processing, motion recall and task switching, while exercising. Thus, TCC is both physical and mental exercise, containing both aerobic exercise and cognitive training (38). Studies have shown that both aerobic exercise and cognitive training can improve cognitive performance (39). In addition, the complex technical movements and organizational forms of TCC are closely related to the updating function of the elderly. This study also showed that compared with controls, subjects who had long-term practice of TCC had faster RTs and higher ARs in performing complex updating tasks. In the practice of TCC, the spatial orientation changes greatly. When an elderly person completes the action of different spatial orientations, they should quickly update the spatial orientation for the next action, to make the correct maneuver, which promotes the development of the updating function. To improve the updating function, it is helpful for elderly people to arrange the whole set of TCC routines on the basis of established movements. They need to combine the whole set of TCC routines based on each one they have learned. When the former movement is completed, it should be updated quickly. This study shows that long-term practice of TCC could improve the performance of the elderly in the N-back tasks, and may be an effective way to maintain the updating function.

TCC involves slow-paced breathing regulation where individuals focus on breathing in and out. In the process of breathing, the mind is directed to intentionally scan the body to influence psychological and physiological states (40). Laborde et al. (41) recruited 78 participants who took part in a 3 × 5 min slow-paced breathing condition and a television viewing control condition. After each condition, heart rate variability was measured and participants performed the color-word match Stroop, the automated operation span task, and the modified card sorting task to test executive function. The results showed that performance on executive function tasks was better after slow-paced breathing compared to control. Slow-paced breathing appears to be a promising technique to improve immediate executive function performance. Another study by Laborde and colleagues (42), suggested that the slow-paced breathing realized before or after physical exertion has a positive effect on adaptation to psychological stress and specifically inhibition. The RTs of the N-back task in this study showed that long-term TCC appears to be associated with better working memory updating function. The TCC practitioners are all the people who have received systematic professional TCC teaching, and have more than 5 years of training experience. They can integrate breathing regulation into daily practice and may obtain psychological and physiological benefits. The electrical activity in the human piriform cortex and limbic-related brain areas are synchronized by natural breathing (43).

TCC contains a meditation component, and studies have shown that meditation improves attention. When Tang et al. (44) recruited 40 subjects for a 5-day meditation intervention experiment, the subjects showed improvements in attention, decreased anxiety, depression, anger, and fatigue, and an increase in immunoreactivity. In addition, TCC is a mindfulness exercise practiced in the natural environment. Research shows that mind-fulness exercise in the natural environment has a stronger effect on attention (45). The improvement of attention also helped the subjects concentrate their cognitive resources to complete the n-back task. This study found that long-term TCC participants were associated with better N-back task performance. TCC requires a relaxed body, calm mind and mind-body unity, and advocates guiding movement with mind (46).

### Limitations of the Study

We would like to acknowledge some limitations of this study. First, this study was a cross-sectional study and could not explain the causal relationship. Second, due to the limitation of experimental conditions, this study only collected behavioral data, and no brain imaging techniques, such as ERP, fNIRS, or fMRI, were utilized. Although some previous neuroimaging studies observed that TCC interventions can lead to changes of brain structure and brain function, there remain open research questions that need to be further explored. For instance, in recent years, multimodal functional neuroimaging has become a hot area of research in neuroscience. Multimodal functional neuroimaging allows for the concurrent assessment of two or more complementary features of brain activity. However, currently and to the best of our knowledge, there are no studies available that used multimodal neuroimaging (e.g., a combination of electroencephalography [EEG] and functional near-infrared spectroscopy [fNIRS]) to investigate the influence of TCC interventions on the human brain. We recommend that future studies use neuroimaging techniques, especially multimodal functional neuroimaging, such as EEG-fNIRS, to investigate the neurobiological processes that drive differences in behavioral performance observed in this study between long-term TCC practitioners and controls.

## Conclusions

In summary, we observed that long-term TCC practitioners reacted faster in the 1-back and 2-back tasks, suggesting that TCC practice can preserve updating function in older adults. Based on our findings and the available literature on TCC and cognition, regular TCC practice can be considered an effective mind-body exercise to preserve cognitive functioning in older adults.

## Data Availability Statement

The original contributions presented in the study are included in the article/supplementary files, further inquiries can be directed to the corresponding author/s.

## Ethics Statement

The studies involving human participants were reviewed and approved by the Capital University of Physical Education and Sports (CUPES) Ethics Committee (approval No. CUPES-2018-06-15-01) approved the study. The patients/participants provided their written informed consent to participate in this study.

## Author Contributions

YY, SY, and CJ: conceptualization and formal analysis. XY, CW, and JZ: software. CJ: resources, project administration, and funding acquisition. YY, TC, CW, JZ, XZ, and XY: data curation. YY: writing-original draft preparation. YY, CW, JZ, SY, XY, XZ, TC, and CJ: writing-review and editing. All authors read and approved the final manuscript.

## Funding

This work was supported by Beijing Natural Science Foundation of China (No. 5212002), the Open Research Fund of the National Center for Protein Sciences at Peking University in Beijing (No. KF-202102), and the National Natural Science Foundation (NNSF) of China under Grant No. 31771244.

## Conflict of Interest

The authors declare that the research was conducted in the absence of any commercial or financial relationships that could be construed as a potential conflict of interest.

## Publisher's Note

All claims expressed in this article are solely those of the authors and do not necessarily represent those of their affiliated organizations, or those of the publisher, the editors and the reviewers. Any product that may be evaluated in this article, or claim that may be made by its manufacturer, is not guaranteed or endorsed by the publisher.

## Abbreviations

TCC: Tai Chi Chuan
RTs: Reaction Times
ARs: Accuracy Rates
WM: Working Memory
MMSE: Minimum Mental State Examination
BMI: Body Mass Index.
